# Supplementary material for: Ecology and evolution of chlamydial symbionts of arthropods
Source: ISME Commun. 2022 May 24;2:45. doi: 10.1038/s43705-022-00124-5 (PMC9723776; doi:10.1038/s43705-022-00124-5)
Supplement: Supplementary file 1 — Supplementary Information [file 43705_2022_124_MOESM1_ESM.pdf]

Supplementary information

Supplementary Text S1: Description of *'Candidatus* Rhabdochlamydia oedothoracis'

[Rhab.do.chla.my'di.a. Gr. fem. n. rhabdos stick, rod; N.L. fem. n. Chlamydia taxonomic name of a bacterial genus; N.L. fem. n. Rhabdochlamydia rod-shaped chlamydiae, referring to the rod-like shape of elementary bodies; oe.do.tho.ra.cis. N.L. gen. n. oedothoracis of Oedothorax, pertaining to the taxonomic genus name of the host organism (a dwarf spider)].

'Candidatus Rhabdochlamydia oedothoracis' infects the spider *Oedothorax gibbosus* [1] and is yet uncultured; its genome sequence was reconstructed from a whole genome sequencing approach of the spider host [2]. Based on its 16S rRNA gene sequence, genomic average nucleotide identities (ANI), and its phylogenetic placement in 16S rRNA and marker gene-based analyses, *'Candidatus* Rhabdochlamydia oedothoracis' (genome sequence accession no. CP075588) belongs to the genus *Rhabdochlamydia* in the family Rhabdochlamydiaceae within the phylum Chlamydiae. *'Candidatus* Rhabdochlamydia oedothoracis' shows a 16S rRNA gene sequence identity of 98 % to *R. porcellionis* (accession no. CP075586) and 98 % to *'Candidatus* Rhabdochlamydia helvetica' (accession no. PRJEB24578). Further, the ANI of *'Candidatus* Rhabdochlamydia oedothoracis' and *'Candidatus* Rhabdochlamydia helvetica' amounts to 88.40 %, and the ANI of *'Candidatus* Rhabdochlamydia oedothoracis' and *R. porcellionis* is 89.85 %.

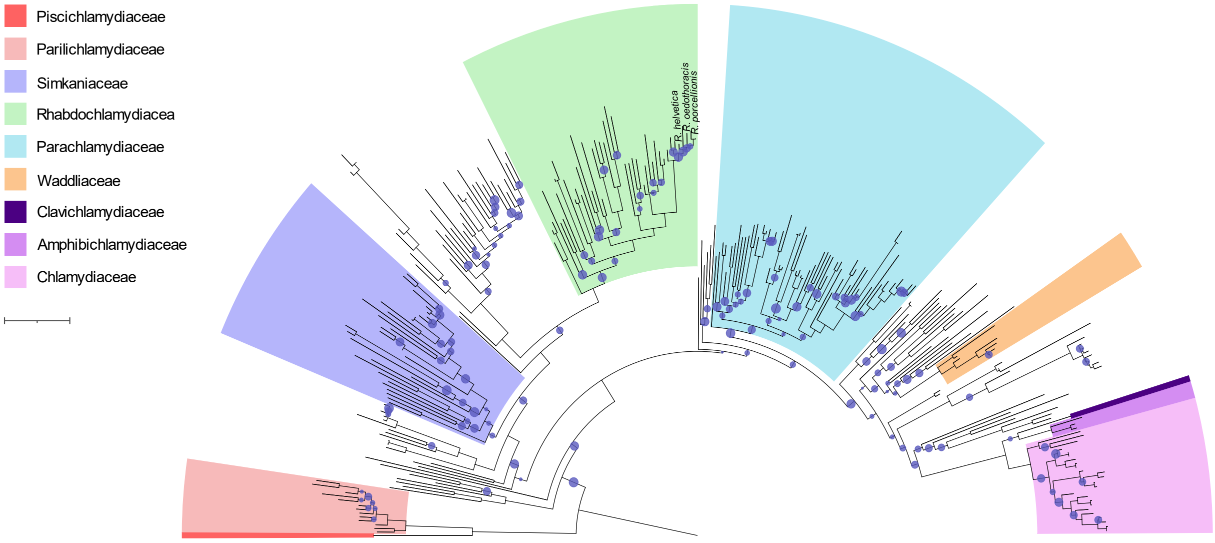

Supplementary figure 1: Full-length 16S tree of the phylum Chlamydiae. Tree is based on full-length alignment (99 % centroids) of 16S sequences retrieved from SILVA, RefSeq, Genbank and Schulz et al. (2017) [3-6]. Tree was rooted using other members of the PVC phylum as an outgroup. Rhabdochlamydiaceae are shown in green. Bootstrap values are depicted by circle sizes (0-0.8). Tree scale: 0.1.

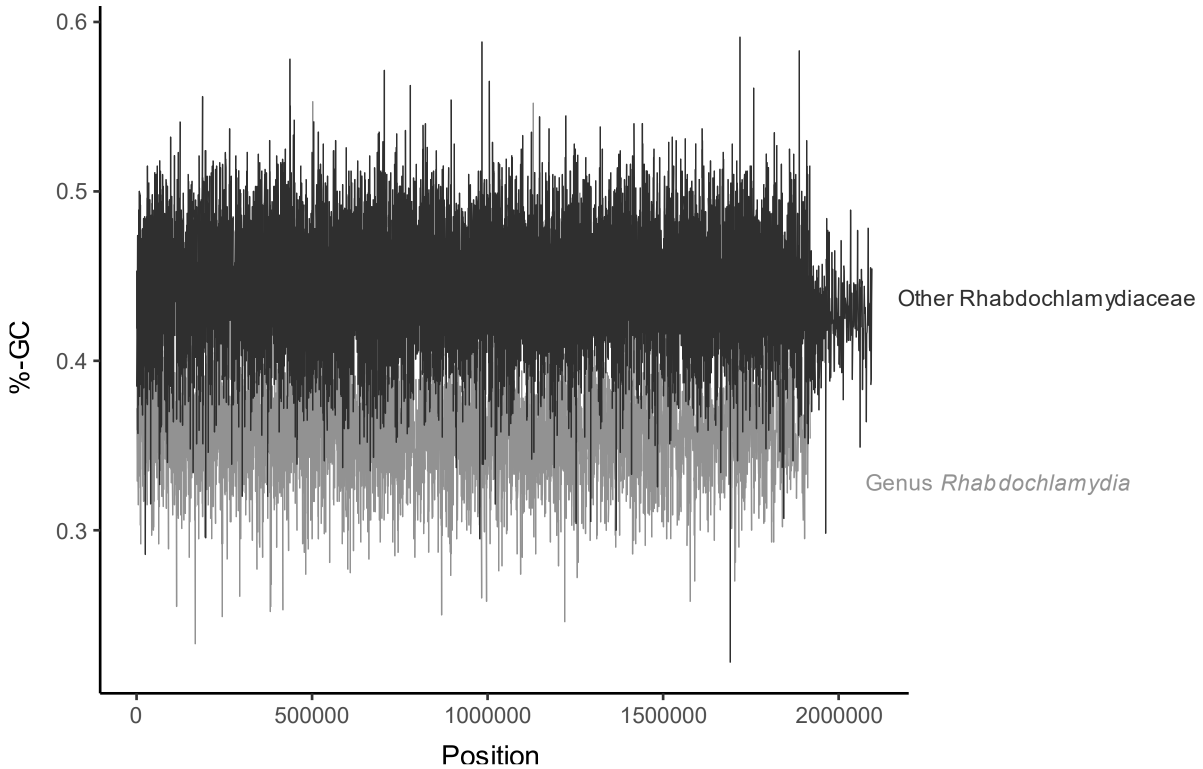

Supplementary figure 2: GC content (window size= 1,000) along rhabdochlamydiae genomes. CG content was calculated using a custom python script. Genomes belonging to the genus *Rhabdochlamydia* (*R. porcellionis*, *R. oedothoracis*, *R. helvetica*) are shown in light grey and three selected MAGs belonging to the family Rhabdochlamydiaceae but not to the genus *Rhabdochlamydia* (1095360-24, 1062783-10, 3300005529-103) are shown in dark grey.

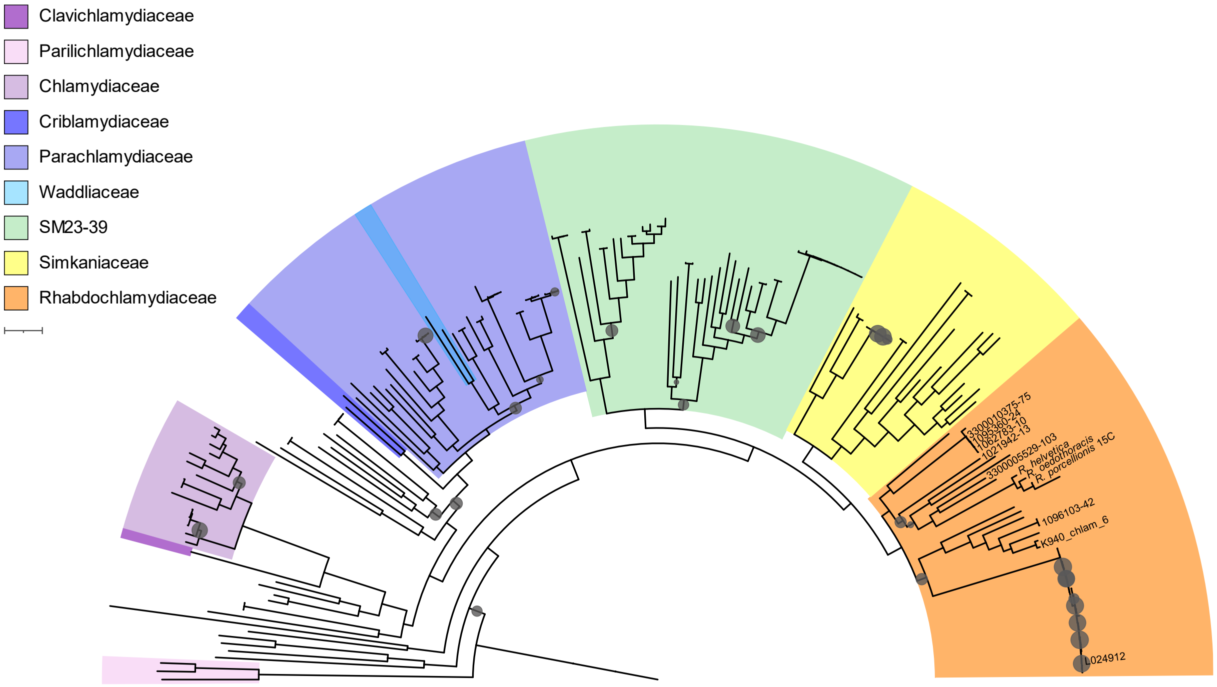

Supplementary figure 3: Marker-gene tree of the phylum Chlamydiae. Rhabdochlamydiaceae are shown in orange. Genomes used in this study are labeled accordingly. Bootstrap values are depicted by circle sizes (0-0.8). Tree scale: 0.1.

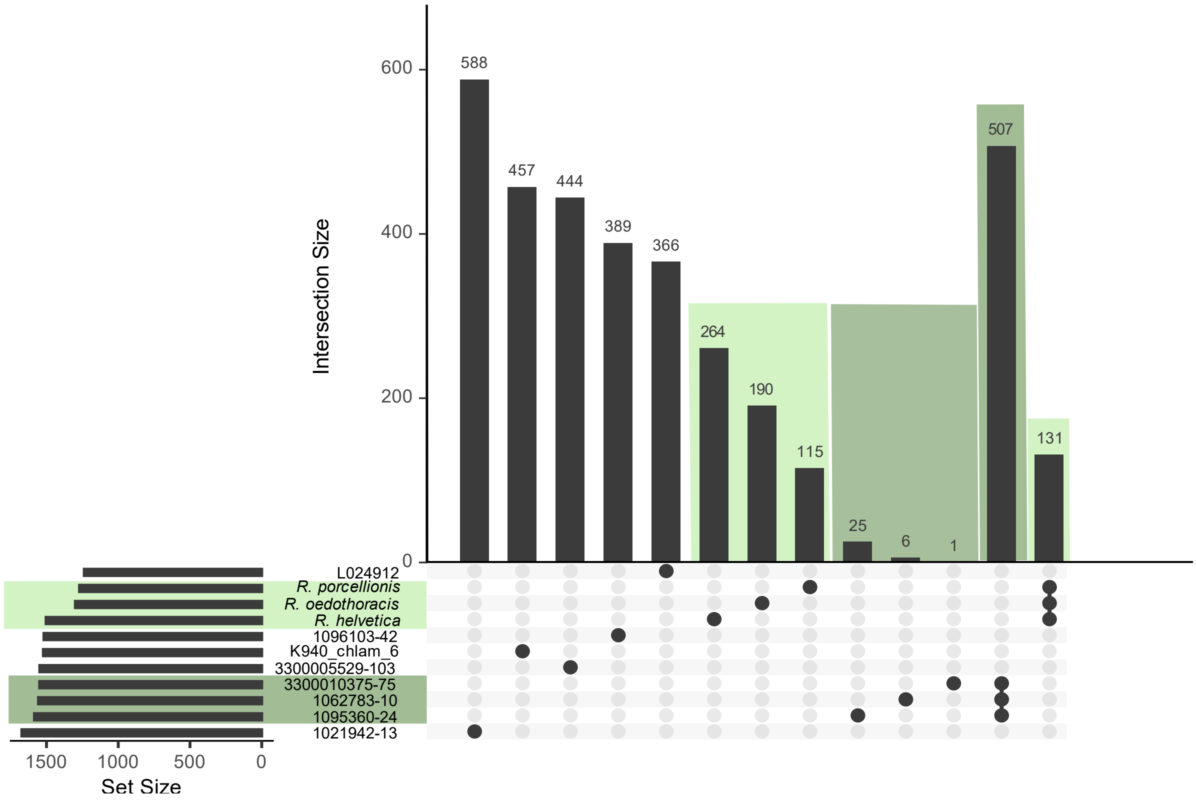

Supplementary figure 4: Pangenome of Rhabdochlamydiaceae. Pangenomes were visualized by UpsetR (v1.4.0) in R (v4.0.3). Genomes belonging to one genus are represented by the same color. Genomes belonging to the genus *Rhabdochlamydia* are colored in light green; another genus consisting of MAGs is labelled in dark green. The set size is the total number of OGs (including eggNOG and de novo clustered OGs) found in a certain genome. Black dots represent genomes and lines represent shared OGs. The intersection size represents the number of OGs occurring in the respective group of genomes.

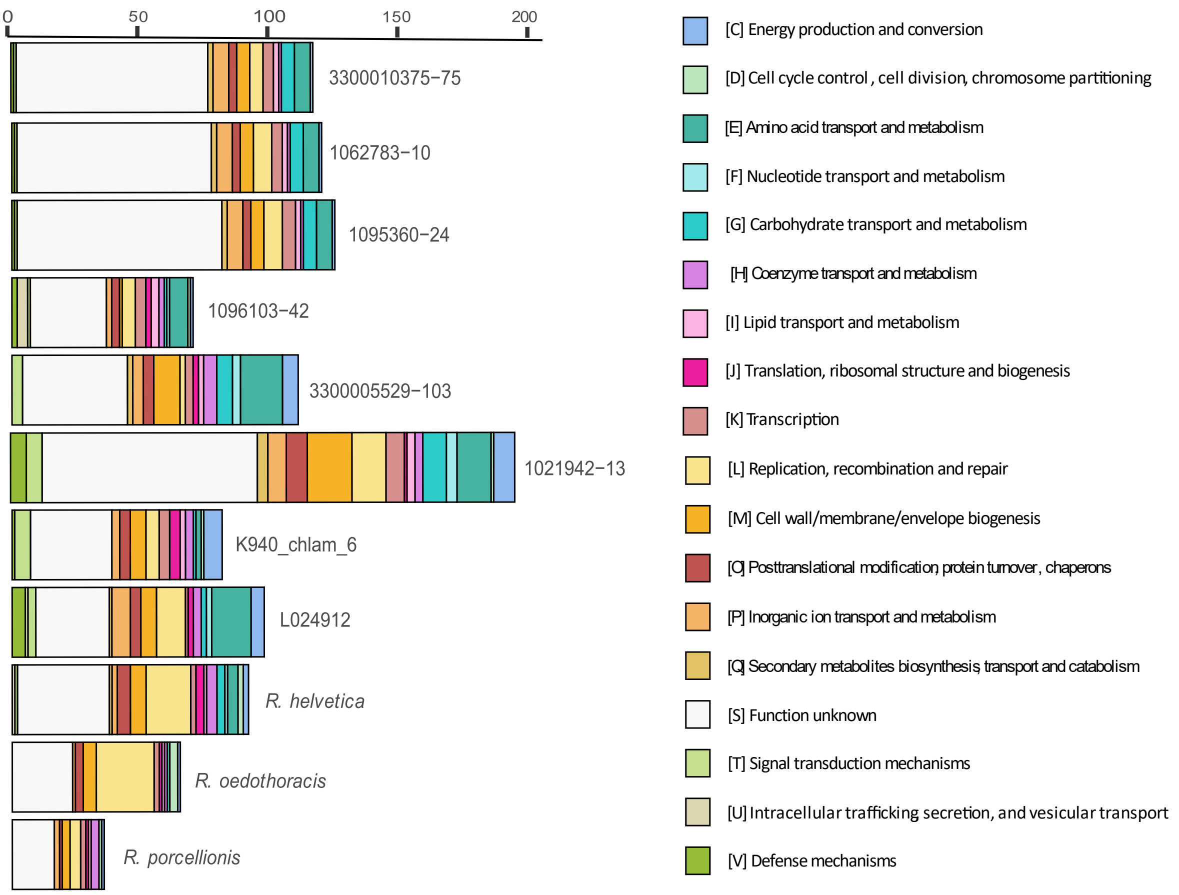

Supplementary figure 5: Stacked-bar chart showing functional categories of rhabdochlamydiae accessory genomes. The analysis only includes genes with a known function. For the plot only unambiguous annotations were used. For genomes belonging to the same genus i.e., the first three and the last three genomes the genus-specific genes were added to the accessory genomes.

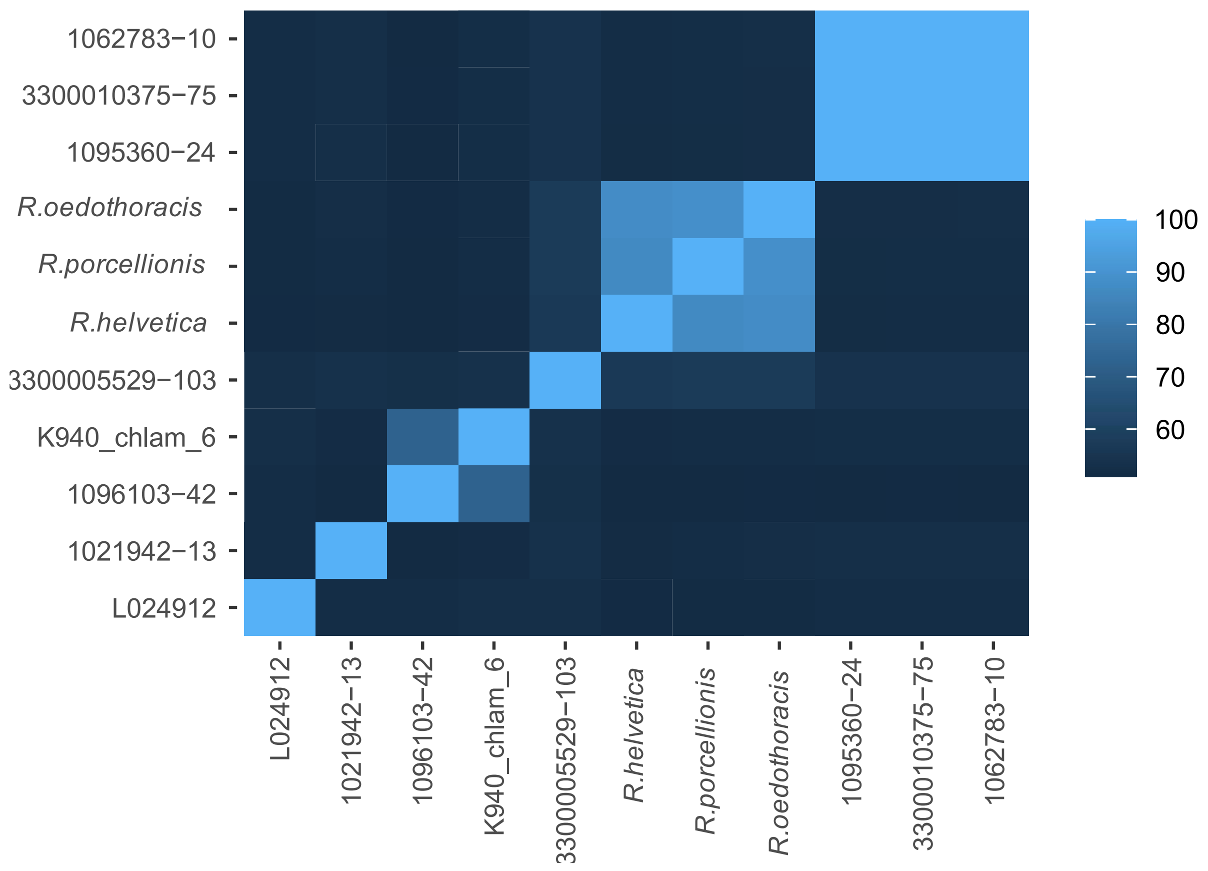

Supplementary figure 6: Average amino acid identity (AAI) of rhabdochlamydiae genomes. The two-way AAI was calculated for each pair of genomes belonging to the family Rhabdochlamydiaceae as described in Konstantinidis et al. (2005) [7]. For all proteins of the query genome a reciprocal best-match was searched in the reference genome. The AAI was calculated considering only those orthologous proteins. We regarded two genomes to belong to one genus if AAI >= 80 %.

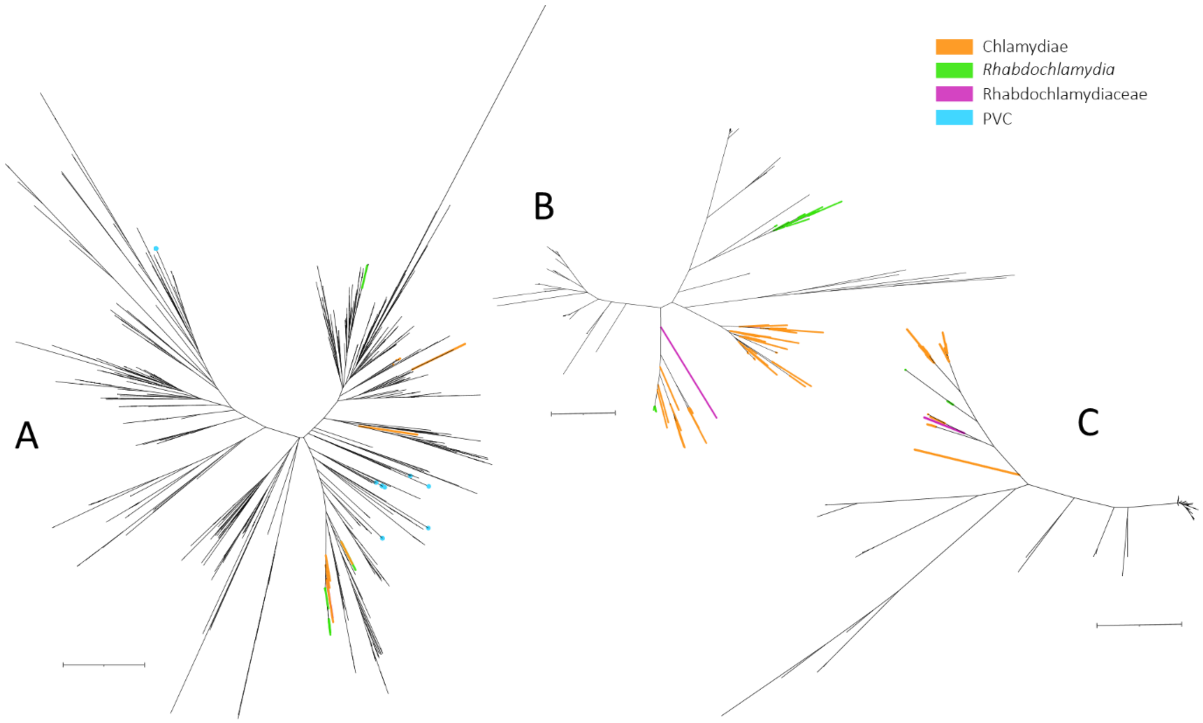

Supplementary figure 7: Phylogeny of the three most abundant functional transposases of *R. oedothoracis*. (**A**) Gene tree for transposase ISRhOegibbosus1 (IS30 family); (**B**) Gene tree for transposase ISRhOegibbosus2 (IS630 family); (**C**) Gene tree for transposase ISRhOegibbosus6 (IS630 family). Chlamydial transposases are indicated by orange, green, and pink branches; blue branches represent homologs from other members of the PVC phylum and black branches represent homologs from other bacteria. Gene trees were calculated as described in Koestlbacher et al. (2021) [8]. Trees were visualized and edited using the Interactive Tree Of Life [9]. As there is no information about the completeness of the transposase genes in the other genomes, the phylogenetic placement may be biased in some cases. Tree scale: 1.

Supplementary references

1. Vanthournout B, Hendrickx F. Endosymbiont dominated bacterial communities in a dwarf spider. *PLoS One* 2015; **10**: e0117297.
2. Hendrickx F, De Corte Z, Sonet G, Van Belleghem SM, Köstlbacher S, Vangestel C. A masculinizing supergene underlies an exaggerated male reproductive morph in a spider. *bioRxiv* 2021; 2021.02.09.430505
3. Quast C, Pruesse E, Yilmaz P, Gerken J, Schweer T, Yarza P, et al. The SILVA ribosomal RNA gene database project: improved data processing and web-based tools. *Nucleic Acids Res* 2013; **41**: D590–6.
4. Haft DH, DiCuccio M, Badretdin A, Brover V, Chetvernin V, O'Neill K, et al. RefSeq: an update on prokaryotic genome annotation and curation. *Nucleic Acids Res* 2018; **46**: D851–D860.
5. Sayers EW, Cavanaugh M, Clark K, Ostell J, Pruitt KD, Karsch-Mizrachi I. GenBank. *Nucleic Acids Res* 2020; **48**: D84–D86.
6. Schulz F, Eloe-Fadrosh EA, Bowers RM, Jarett J, Nielsen T, Ivanova NN, et al. Towards a balanced view of the bacterial tree of life. *Microbiome* 2017; **5**: 140.
7. Konstantinidis KT, Tiedje JM. Towards a genome-based taxonomy for prokaryotes. *J Bacteriol* 2005; **187**: 6258–6264.
8. Köstlbacher S, Collingro A, Halter T, Schulz F, Jungbluth SP, Horn M. Pangenomics reveals alternative environmental lifestyles among chlamydiae. *Nat Commun* 2021; **12**: 4021.
9. Letunic I, Bork P. Interactive Tree Of Life (iTOL) v4: recent updates and new developments. *Nucleic Acids Res* 2019; **47**: W256–W259.
