# Supplementary material for: Ecology and evolution of chlamydial symbionts of arthropods
Source: ISME Commun. 2022 May 24;2:45. doi: 10.1038/s43705-022-00124-5 (PMC9723776; doi:10.1038/s43705-022-00124-5)
Supplement: Supplementary file 2 — Supplementary tables [file 43705_2022_124_MOESM2_ESM.pdf]

Supplementary table S1: Basic dataset info.

Name	Identifier	Accession	IMG genome ID	CDS	GC [%]	Genome size [bp]	Family	Completness [%]	Heterogeneity [%]	Contamination [%]
Chlamydia abortus S26/3	CHLAB	GCF_000026025.1		935	39.87	1144377	Chlamydiaceae	98.28	0.00	0.00
Chlamydia ibidis 10-1398/6	CHLAIB	GCF_000454725.1		939	38.32	1146066	Chlamydiaceae	94.83	0.00	0.00
Chlamydia sp. RSHA	CHLBUT	GCF_900634605.1		942	38.29	1146546	Chlamydiaceae	98.28	0.00	0.00
Candidatus Chlamydia corallus strain G3/2742-324	CHLCOR	GCF_002817655.1		1016	39.28	1203974	Chlamydiaceae	98.28	0.00	0.00
Chlamydophila caviae GPIC	CHLCV	GCF_000007605.1		976	39.19	1181356	Chlamydiaceae	98.28	0.00	0.00
Chlamydia felis Fe/C-56	CHLFF	GCF_000009945.1		974	39.34	1173791	Chlamydiaceae	98.28	0.00	0.00
Chlamydia gallinacea 08-1274/3	CHLGAL	GCF_000471025.2		912	37.9	1067202	Chlamydiaceae	94.83	0.00	0.00
Chlamydia muridarum str. Nigg	CHLMU	GCF_000006685.1		908	40.31	1080451	Chlamydiaceae	98.28	0.00	0.00
Chlamydia psittaci 6BC	CHLP6	GCF_000204255.1		988	39.02	1179220	Chlamydiaceae	98.28	0.00	0.00
Chlamydophila pecorum E58	CHLPE	GCF_000204135.1		934	41.08	1106197	Chlamydiaceae	100.00	0.00	0.00
Chlamydophila pneumoniae CWL029	CHLPN	GCF_000008745.1		1029	40.58	1230230	Chlamydiaceae	98.28	0.00	0.00
Chlamydophila pneumoniae LPCoLN	CHLPP	GCF_000024145.1		1105	40.5	1248550	Chlamydiaceae	98.28	0.00	0.00
Chlamydia sp. 2742-308	CHLSAN	GCF_001653975.1		940	38.5	1120737	Chlamydiaceae	98.28	0.00	0.00
Chlamydia suis MD56	CHLSUIS	GCF_000493885.1		892	42.01	1079129	Chlamydiaceae	98.28	0.00	0.00
Chlamydia trachomatis 434/Bu	CHLT2	GCF_000068585.1		880	41.33	1038842	Chlamydiaceae	98.28	0.00	0.00
Chlamydia trachomatis A/HAR-13	CHLTA	GCF_000012125.1		900	41.27	1051969	Chlamydiaceae	96.55	0.00	0.00
Chlamydia trachomatis D/UW-3/CX	CHLTR	GCF_000008725.1		887	41.31	1042519	Chlamydiaceae	96.55	0.00	0.00
1041544-29	1041544-29		1041544	2382	38.09	2850003	Parachlamydiaceae	98.28	0.00	0.00
3300009084-136	3300009084-136		3300009084	1941	34.86	2250271	Parachlamydiaceae	96.55	0.00	0.00
3300009084-150	3300009084-150		3300009084	1747	45.72	1750468	Parachlamydiaceae	100	0.00	0.00
Protochlamydia sp. ACF82	ACE82	JAEMUB000000000		2233	34.49	2331284	Parachlamydiaceae	95.69	0.00	0.00
Parachlamydia sp. ACF125	ACF125	JAEMUD000000000		1919	40.77	2584652	Parachlamydiaceae	98.28	0.00	0.00
Chlamydia sp. 32-24	CHL3224	GCA_001897185.1		2075	32.42	2529957	Parachlamydiaceae	98.28	0.00	0.00
Chlamydiales bacterium 38-26	CHL3826	GCA_001897225.1		2327	38.12	2834110	Parachlamydiaceae	98.28	0.00	0.00
Protochlamyida sp. EI2	EI2	GCA_000813625.1		2150	34.82	2397675	Parachlamydiaceae	96.55	0.00	0.00
Neochlamydia sp. EPS4	EPS4	GCF_000813665.1		1882	38.09	2530677	Parachlamydiaceae	96.55	0.00	0.00
Chlamydiales bacterium STE3	HSC3	VKHO00000000		2176	39.17	2231767	Parachlamydiaceae	98.28	0.00	0.00
Parachlamydia acanthamoebae OEW1	OEW1	GCA_000812225.1		2309	39.04	3008885	Parachlamydiaceae	94.83	0.00	0.00
Parachlamydia acanthamoebae UV-7	PARAV	GCF_000253035.1		2532	39.04	3072383	Parachlamydiaceae	96.55	0.00	0.00
Candidatus Protochlamydia amoebophila UWE25	PARUW	GCF_000011565.1		1841	34.72	2414465	Parachlamydiaceae	98.28	0.00	0.00
Parachlamydia sp. C2	PROGREC2	GCA_001545115.1		2766	42.05	3424182	Parachlamydiaceae	100.00	0.00	0.00
Protochlamydia naegleriophila	ProNeg	GCA_001499655.1		2520	42.44	3030375	Parachlamydiaceae	100.00	0.00	0.00
Protochlamydia massiliensis	PRONEGDIA	GCF_000751535.1		2451	42.75	2956128	Parachlamydiaceae	98.28	0.00	0.00
Parachlamydia sp. isolate BC.030	PROPHO	GCA_002786175.1		2540	41.53	3042961	Parachlamydiaceae	100.00	0.00	0.00
Rubidus massiliensis	Rubis	GCA_000756735.1		2446	32.64	2821221	Parachlamydiaceae	98.28	0.00	0.00
Neochlamydia sp. TUME1	TUME1	GCF_000813645.1		1879	38.02	2546323	Parachlamydiaceae	96.08	0.00	0.00
1021942-13	1021942-13		1021942	1792	42.3	2050771	Rhabdochlamydiaceae	97.41	0.00	0.00
1062783-10	1062783-10		1062783	1724	45.19	1859781	Rhabdochlamydiaceae	100	0.00	0.00
1095360-24	1095360-24		1095360	1750	45.12	1889532	Rhabdochlamydiaceae	100	0.00	0.00
1096103-42	1096103-42		1096103	1637	44.67	1644418	Rhabdochlamydiaceae	95.69	0.00	0.00
3300005529-103	3300005529-103		3300005529	1695	43.86	1937521	Rhabdochlamydiaceae	100	0.00	0.00
3300010375-75	3300010375-75		3300010375	1704	45.18	1846245	Rhabdochlamydiaceae	94.59	0.00	0.00
Chlamydiae bacterium K940_chlam_6	K940_chlam_6	GCA_001796315.1		1750	42.93	1505150	Rhabdochlamydiaceae	95.69	0.00	0.00
Chlamydiae bacterium RIFCSPLOWO2_02_FULL_49_12	L024912	from PMID: 32142706		1313	48.99	1413529	Rhabdochlamydiaceae	96.55	0.00	0.00
Rhabdochlamydia helvetica	RhabHel	from PMID: 30949677		1717	36.2	1854477	Rhabdochlamydiaceae	94.59	0.00	0.00

Supplementary table S2: Basic genome statistics of complete *Rhabdochlamydia* genomes.

	***R. porcellionis* 15C**	***R. oedothoracis* W744**
Closed	yes	yes
Estimated size [mb]	1.49	1.88
Plasmid [kb]	19.7	38.9
Contigs	2	2
Completeness [%]	100	100
Contamination [%]	0	0
Heterogeneity [%]	0	0
tRNA	37	37
rRNA	6	6
CDS	1.348	1.569
GC [%]	35.4	36.2

Supplementary table S3: OGs discriminating the genus *Rhabdochlamydia* from other members of the family Rhabdochlamydiaceae

OG missing in genomes of the family Rhabdochlamydiaceae but present in all members of the genus *Rhabdochlamydia*	**Functional Category**	**Membrane [Y/N]**	**Description**	**Evidence**
05CPH	H	N	Catalyzes the formation of S-adenosylmethionine from methionine and ATP	
05DV8	S	N	filamentation induced by cAMP protein Fic	
05EGX	M	Y	Nad-dependent epimerase dehydratase	Functional Category
05ERD	L	N	Involved in base excision repair of DNA damaged by oxidation or by mutagenic agents. Acts as DNA glycosylase that recognizes and removes damaged bases. Has a preference for oxidized purines, such as 7,8-dihydro-8-oxoguanine (8-oxoG). Has AP (apurinic apyrimidinic) lyase activity and introduces nicks in the DNA strand. Cleaves the DNA backbone by beta-delta elimination to generate a single-strand break at the site of the removed base with both 3'- and 5'-phosphates (By similarity)	
05F0X	D	N	Cobyrinic acid ac-diamide synthase	
05F23	K	Y	Fibronectin-binding protein	https://doi.org/10.1007/s00430-019-00644-3
05RKK	S	N	Domain of unknown function (DUF202)	
05Z3Q	J	N	tRNA rRNA methyltransferase	
06A7V	S	Y	Permease of the drug metabolite transporter DMT superfamily	GO Term: Cellular Component
06VZX	L	N	establishment of viral latency	
071KN	S	Y	MOMP-like family	https://doi.org/10.1128/IAI.69.5.3082-3091.2001
07EW0	M	Y	peptidase	Functional Category
07GNU	C	Y	TLC ATP/ADP transporter	GO Term: Cellular Component
07SJN	I	N	Catalyzes the phosphorylation of the position 2 hydroxy group of 4-diphosphocytidyl-2C-methyl-D-erythritol (By similarity)	
07WZ0	P	N	ferritin dps family protein	
085BK	S	N	UPF0109 protein	
08JHT	E, G	Y	EamA-like transporter family	GO Term: Cellular Component
08MMU	S	N	Pfam:DUF2843	
08NMA	L	N	Plasmid and phage replicative helicase	
08SEI	S	N	SOUL heme-binding protein	
0NHB5	O	N	PPIases accelerate the folding of proteins	
0QNFT	M	Y	glycosyl transferase group 1	Functional Category
05ZQP	S	N	NA	
060UR	S	N	NA	

OG present in all genomes of the family Rhabdochlamydiaceae but completely missing in the genus *Rhabdochlamydia*	Functional Category	Membrane [Y/N]	Description	Evidence
05C5C	M	Y	n-acetylmuramoyl-l-alanine amidase	Functional Category
05C7Z	O	N	Conserved Protein	
05DCI	S	N	Phosphohydrolase	
05DKP	M	Y	glycosyl transferase, family 9	Functional Category
05VH8	J	N	Binds as a heterodimer with protein S6 to the central domain of the 16S rRNA, where it helps stabilize the platform of the 30S subunit (By similarity)	
07NVQ	N	Y	Flagellar biosynthesis protein, FliO	Functional Category
07RI4	M	Y	Transfers the fatty acyl group on membrane lipoproteins (By similarity)	Functional Category
08RMU	P	N	ferritin dps family protein	

Supplementary table S4: Overview of transposases of *R. porcellionis* and *R. oedothoracis* . For each transposon the counts of functional genes and pseudogenes are depicted. The length always refers to functional genes.

Organism	Name, IS group/family	# pseudogenes	# functional genes	# total	Length [nt]	Plasmid [Y/N]
R. oedothoracis	Transposase ISRhOegibbosus1, IS30 family	25	74	99	1.845	Y
R. oedothoracis	Transposase ISRhOegibbosus2, IS630 family	42	42	84	1.035	Y
R. oedothoracis	Transposase ISRhOegibbosus3, IS1031 group IS5 family	79	0	79		N
R. oedothoracis	Transposase ISRhOegibbosus4, IS1634 family	3	0	3		N
R. oedothoracis	Transposase ISRhOegibbosus5, IS427 group IS5 family	32	0	32		Y
R. oedothoracis	Transposase ISRhOegibbosus6, IS630 family	31	9	40	804	Y
R. oedothoracis	Transposase ISRhOegibbosus7, IS110 group IS1111	18	0	18		N
R. oedothoracis	Transposase ISRhOegibbosus8, ISL2 group IS5 family	17	0	17		N
R. oedothoracis	Transposase ISRhOegibbosus9, IS630 family	11	2	13	1.032	N
R. oedothoracis	Transposase ISRhOegibbosus10, IS481 family	12	0	12		Y
R. oedothoracis	Transposase ISRhOegibbosus11, IS982 family	3	0	3		N
R. oedothoracis	Transposase ISRhOegibbosus12, ISAs1 family	2	0	2		N
R. oedothoracis	Transposase ISRhOegibbosus13, IS630 family	3	0	3		N
R. oedothoracis	Transposase ISRhOegibbosus14, ISAs1 family	7	1	8	1.143	N
R. oedothoracis	Transposase ISRhOegibbosus15, IS3 group IS3 family	1	0	1		N
R. oedothoracis	Transposase ISRhOegibbosus16, IS481 family	1	0	1		N
R. procellionis	Transposase ISRhPorc1, ISL2 group IS5 family	2	0	2		N
R. procellionis	Transposase ISRhPorc2, IS630 family	12	0	12		N
R. procellionis	Transposase ISRhPorc3, ISL2 group IS5 family	5	0	5		N
R. procellionis	Transposase, PD-(D/E)XK nuclease family	0	1	1	936	N
